# Spectra of weighted scale-free networks

**DOI:** 10.1038/srep17469

**Published:** 2015-12-04

**Authors:** Zhongzhi Zhang, Xiaoye Guo, Yuhao Yi

**Affiliations:** 1 School of Computer Science, Fudan University, Shanghai 200433, China; 2 Shanghai Key Laboratory of Intelligent Information Processing, Fudan University, Shanghai 200433, China

## Abstract

Much information about the structure and dynamics of a network is encoded in the eigenvalues of its transition matrix. In this paper, we present a first study on the transition matrix of a family of weight driven networks, whose degree, strength, and edge weight obey power-law distributions, as observed in diverse real networks. We analytically obtain all the eigenvalues, as well as their multiplicities. We then apply the obtained eigenvalues to derive a closed-form expression for the random target access time for biased random walks occurring on the studied weighted networks. Moreover, using the connection between the eigenvalues of the transition matrix of a network and its weighted spanning trees, we validate the obtained eigenvalues and their multiplicities. We show that the power-law weight distribution has a strong effect on the behavior of random walks.

As a standard tool, random walks on a network describes various dynamical processes in the network, such as search^1^,^2^ spreading^3^, diffusion^4^, to name a few. Due to its role as a fundamental mechanism characterizing diverse other processes, random walks on complex networks have attracted considerable attention in the past years^5^,^6^,^7^,^8^,^9^,^10^,^11^,^12^,^13^,^14^,^15^,^16^,^17^,^18^,^19^. The vast literature provided novel methods for computing mean first-passage time, steady-state distribution, as well as many other properties of random walks.

Since random walks are completely described by the transition matrix^20^, most interesting quantities and properties related to random walks are determined by the spectra (eigenvalues and eigenvectors) of the transition matrix. First of all, the mean first-passage time from one node to another can be represented through the eigenvalues and eigenvectors of the transition matrix^20^. Furthermore, the sum of reciprocals of one minus every eigenvalue, excluding the eigenvalue 1 itself, determines the random target access time^21^. Finally, the smallest eigenvalue, together with the second largest eigenvalue, provides an upper bound and a lower bound for the mixing time^22^. In addition to the properties of random walks, the spectra of the transition matrix for a network are also pertaining to structural aspects of the network, for example, spanning trees^23^,^24^ and effective resistance^25^, which can also be determined by the spectra of Laplacian matrix^26^. Thus, transition matrix is closely related to Laplacian matrix, with the latter being widely used in quantum walks^27^,^28^ and quantum algorithms^29^.

In view of the significance, the study of spectra for transition matrix has become a central issue^30^. In the past decade, there has been important progress in determining the eigenvalues for transition matrix of different networks or characterizing their properties. Examples include random graphs^31^,^32^, Sierpinski gasket^33^,^34^, Tower of Hanoi graph^35^, Cayley tree and extend T-fractal^36^, fractal^37^,^38^ and non-fractal^39^,^40^ scale-free networks. These works provided a deeper understanding on spectral characteristics of the transition matrix of different networks, as well as the effects of network topology on the spectral density and random-walk process. Extensive empirical research has unveiled that real networks are characterized by the heterogeneity^41^,^42^, not only in the aspect of degree distribution^43^ but also in the context of weight distribution^44^,^45^,^46^. Previous works about spectra of the transition matrix were limited to binary networks, and the influence of inhomogeneous weight distribution on the spectral properties of transition matrix still remains unknown.

In this paper, we study analytically the eigenvalues for transition matrix of a class of weighted networks^47^, which exhibit some prominent properties that are observed in real-world systems^44^,^45^,^46^, such as the power-law distribution of node degree, strength, and edge weight. Based on the particular construction of the networks, we find all the eigenvalues and their corresponding multiplicities. Using the obtained eigenvalues, we deduce an explicit expression for the random target access time, as well as its leading scaling, which is different from those previously obtained for binary heterogeneous networks, implying that the weight has an important impact on the random-walk behavior. Moreover, we determine the weighted counting of spanning trees in the studied networks using the eigenvalues, which is consistent with that derived by another technique, corroborating the validity of our computation for the eigenvalues.

## Results

### Construction and properties of weight driven scale-free weighted networks

**Figure 1:**
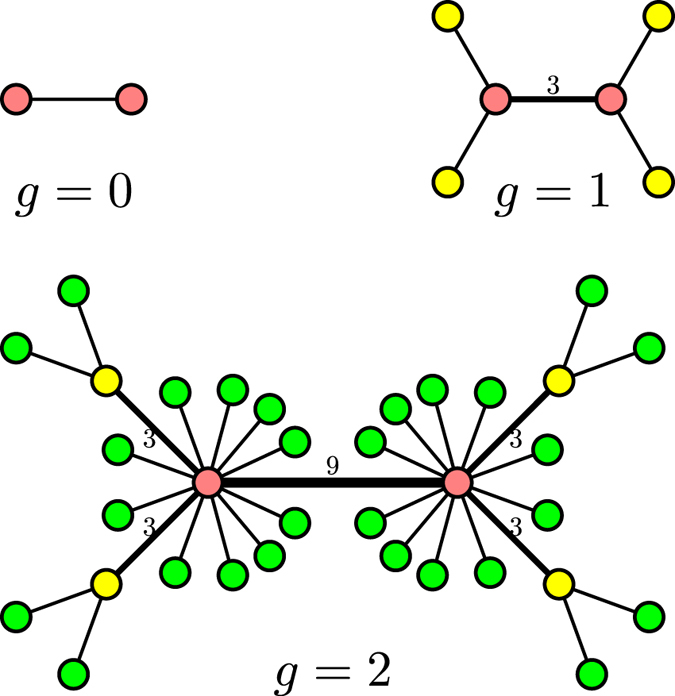
Illustration of the growth for a particular network. The growth process corresponds to *m* = 2 and *δ* = 1, showing the first three iterations. The bare edges denote those edges of unit weight.

The network family, parameterized by two positive integer *m* and *δ*, is constructed in an iterative manner^47^. Let 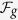 denote the network class after *g* (*g* ≥ 0) iterations. Then, the network family is built as follows. For *g* = 0, 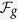 consists of an edge (link) with unit weight connecting two nodes (vertices). For *g* ≥ 1, 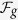 is obtained from 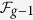 by performing the following operations. For each edge with weight *w* in 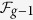, we add *mw* new nodes for either end of the edge and connect each of the *mw* new nodes to the end by new edges of unit weight; then we increase weight *w* of the edge by *mδw*. Figure 1 illustrates the network generation process for a special case of *m* = 2 and *δ* = 1.

Let *N*_*g*_, *E*_*g*_, *Q*_*g*_ denote, respectively, the total number of nodes, the total number of edges, and the total weight of all edges in 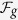. And let *n*_*v*_(*g*) and *n*_*e*_(*g*) denote, respectively, the number of nodes and the number of edges that are created at iteration *g*. Then, *n*_*e*_(*g*) = *n*_*v*_(*g*) holds for all *g* ≥ 1. By construction, for *g* ≥ 0, we have


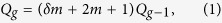


which under the initial condition *Q*_0_ = 1 yields


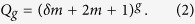


Furthermore, it is easy to derive that for all *g* ≥ 1,


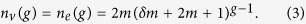


Thus,


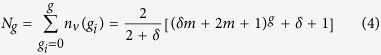


and


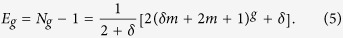


For an edge *e* connecting two nodes *i* and *j* in 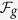, which is born at iteration *τ*, we use *w*_*e*_(*g*) or *w*_*ij*_(*g*) to denote its weight. Then, *w*_*e*_(*g*) = *w*_*ij*_(*g*) = (*δm* + 1)^*g*−*τ*^. Let *s*_*i*_(*g*) (resp. *d*_*i*_(*g*)) be the strength (resp. degree) of node *i* in 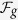, which is added to the network at generation *g*_*i*_. It is easy to obtain


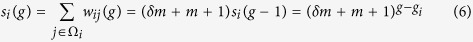


and


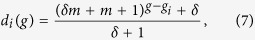


where Ω_*i*_ is the set of neighbors of *i* in 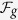.

The resultant networks display prominent properties as observed in real systems^44^,^45^,^46^, with their degree, strength, and edge weight following power law distribution^47^.

### Eigenvalues and multiplicities of transition matrix

After introducing the construction and properties of the weighted scale-free networks, in this section we study eigenvalues and their multiplicities of the transition matrix for the networks.

#### Recursive relation of eigenvalues

Let **W**_*g*_ be the generalized adjacency matrix (weight matrix) of 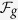. The entries *W*_*g*_(*i*, *j*) of **W**_*g*_ are defined as follows: 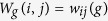 if nodes *i* and *j* are adjacent in 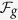, or 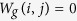 otherwise. Then, the transition matrix for biased random walks^48^,^49^ in 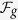, denoted by **T**_*g*_, is defined as 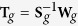, where **S**_*g*_ is the diagonal strength matrix of 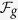 with its *i*th diagonal entry being the strength *s*_*i*_(*g*) of node *i*. Thus, the (*i*, *j*)th element of **T**_*g*_ is 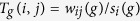, which represents the local transition probability for a walker going from node *i* to node *j*.

We now consider the eigenvalues and eigenvectors of **T**_*g*_. Since **T**_*g*_ is asymmetric, we introduce the following real and symmetric matrix **P**_*g*_ defined as


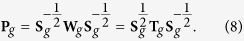


By definition, the (*i*, *j*)th entry of **P**_*g*_ is 
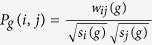
. Since **P**_*g*_ is similar to **T**_*g*_, they have the same set of eigenvalues. Furthermore, if *ϕ* is an eigenvector of matrix **P**_*g*_ associated with eigenvalue *λ*, then 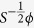 is an eigenvector of **T**_*g*_ corresponding to eigenvalue *λ*. Therefore, we reduce the problem of finding eigenvalues for an asymmetric matrix **T**_*g*_ to the issue of determining eigenvalues for a symmetric matrix **P**_*g*_.

Suppose that *λ* is an eigenvalue of **P**_*g*_, and 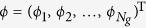 is its corresponding eigenvector, where *ϕ*_*j*_ is the component corresponding to node *j* in 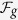. Let 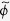 be a vector of dimension *N*_*g*−1_ that is obtained from *ϕ* by restricting its components to the old nodes, namely, nodes generated before or at iteration *g* − 1. As will be shown below, 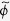 is an eigenvector of **P**_*g*−1_, associated with eigenvalue 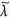, from which *λ* is generated. By definition, we have


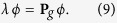


Let *o* be an old node in 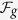. According to Eq. (9),


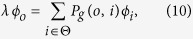


where Θ denotes the set of the *d*_*o*_(*g*) neighbors of node *o*. Let 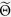 be the set of the *d*_*o*_(*g* − 1) old neighbors of node *o*, while the other new neighbors form set 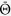. For each new neighboring node 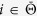, one has 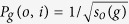, which implies 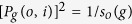. Thereby, the component *ϕ*_*i*_ satisfies


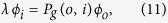


implying


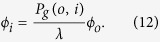


In the case *λ*≠0, inserting Eq. (12) into Eq. (10) and considering the two relations 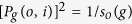 and 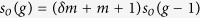, we obtain


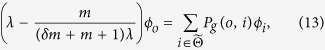


an equation only involving old nodes, which were already existing at iteration *g* − 1.

For *λ*≠0, Eq. (13) is true for an arbitrary node present at generation *g* − 1. Thus, we can compare Eq. (13) with the following corresponding equation for the old node *o* at iteration *g* − 1:


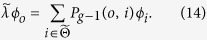


This indicates that 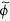 is an eigenvector of **P**_*g*−1_, corresponding to eigenvalue 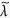.

It is not difficult to see that the entry *P*_*g*−1_(*o*, *i*) of generation *g* − 1 is 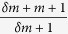 times its counterpart *P*_*g*_(*o*, *i*) of generation *g*. Then, Eqs. (13) and (14) coincide, provided that


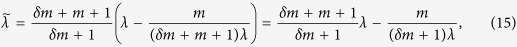


which relates *λ* to 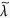. Solving the quadratic equation in the variable *λ* given by Eq. (15) yields


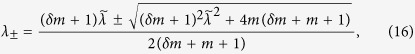


which shows that each eigenvalue 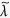 of **P**_*g*−1_ gives rise to two eigenvalues of **P**_*g*_, *λ*_+_ and *λ*_−_.

Let *ϕ*^+^ and *ϕ*^−^ denote the eigenvectors of *λ*_+_ and *λ*_−_, respectively. Then, both *ϕ*^+^ and *ϕ*^−^ can be obtained from 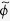 in the following way. For the nodes already present at iteration *g* − 1, the components of *ϕ*^+^ and *ϕ*^−^ are equivalent to the corresponding components of 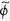; while for the nodes generated at iteration *g*, their components can be determined by replacing *λ* in Eq. (12) with *λ*_+_ or *λ*_−_. Therefore, *λ*_+_ (or *λ*_−_) has the same number of linearly independent eigenvectors as that of 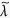. Moreover, the eigenvectors of *λ*_+_ (or *λ*_−_) are linearly independent, because **P**_*g*_ is real and symmetric.

### Multiplicities of eigenvalues

Equation (16) indicates that from the eigenvalues of generation *g* − 1, one can obtain the eigenvalues of the next generation *g*, with the exception of those zero eigenvalues. Thus, if there exists an eigenvalue *λ* that cannot be derived from Eq. (16), it must be zero eigenvalue. Let 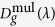 represent the degeneracy of eigenvalue *λ* for matrix **P**_*g*_. Because **P**_*g*−1_ is a real and symmetrical matrix, each eigenvalue 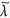 of **P**_*g*−1_ has 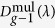 linearly independent eigenvectors. It is the same with either of its child eigenvalues, *λ*_+_ or *λ*_−_. Next we determine the multiplicity of each eigenvalue for matrix **P**_*g*_.

For small networks, the eigenvalues and their multiplicities can be calculated directly. The eigenvalues of **P**_0_ are 1 and −1. For **P**_1_, its eigenvalues are 1, −1, 0, 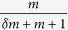, and 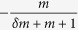, where two pairs of eigenvalues (1 and 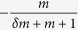, −1 and 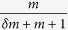) are generated, respectively, by eigenvalues 1 and −1 of **P**_0_. Moreover, the offspring eigenvalue of 1 and −1 has a single degeneracy. For *g* ≥ 2, the eigenvalues of matrix **P**_*g*_ display the following remarkable nature. Every eigenvalue appearing at current generation *g*_*i*_ always exists at the next generation *g*_*i*_ + 1, and all new eigenvalues of 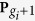 are those produced via Eq. (16) by substituting 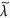 with those eigenvalues that were newly borne at generation *g*_*i*_; moreover every new eigenvalue inherits the multiplicity of its parent. Hence, for *g* ≥ 2, all eigenvalues (excluding zero eigenvalue) of **P**_*g*_ are generated from 1, −1, and 0, with all the offspring eigenvalues of 1 and −1 being nondegenerate. Therefore, all that is left is to determine the multiplicity of 0, as well as the multiplicities of its descendants.

Let *r*(**M**) denote the rank of matrix **M**. Then, the multiplicity of the zero eigenvalues for **P**_*g*_ is


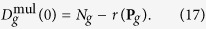


We now evaluate *r*(**P**_*g*_). For the set of all nodes in 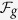, let *α* denote the subset of nodes in 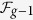, and *β* the subset of nodes newly produced at generation *g*. Then, **P**_*g*_ can be written in a block form


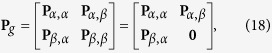


where the fact that **P**_*β*,*β*_ is the (*N*_*g*_ − *N*_*g*−1_) × (*N*_*g*_ − *N*_*g*−1_) zero matrix is used.

Notice that *r*(**P**_*α*,*β*_) = *r*(**P**_*β*,*α*_). We can prove that (see Methods) **P**_*β*,*α*_ is a full column rank matrix. Then, 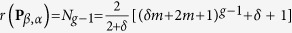 and 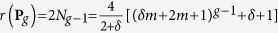. According to Eq. (17), the multiplicity of eigenvalue 0 for matrix **P**_*g*_ is: 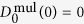 for *g* = 0; and 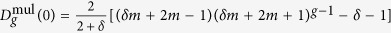 for *g* ≥ 1. Because each eigenvalue in **P**_*g*_ keeps the degeneracy of its parent, the number of each of the first-generation descendants of zero eigenvalue is 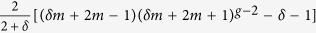, the number of each of the second-generation descendants of zero eigenvalue is 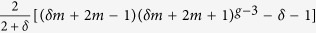, and so on. Thus, the total number of zero eigenvalue and its descendants in **P**_*g*_ (*g* ≥ 1) is


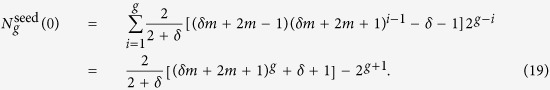


For eigenvalue 1 (or −1), the total number of its descendants in **P**_*g*_ (*g* ≥ 0), including 1 (or −1) itself, is


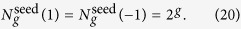


Adding up the number of the above-obtained eigenvalues yields


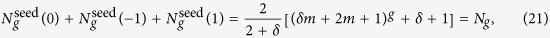


which implies that we have found all the eigenvalues of matrix **P**_*g*_ and thus the transition matrix **T**_*g*_.

**Figure 2:**
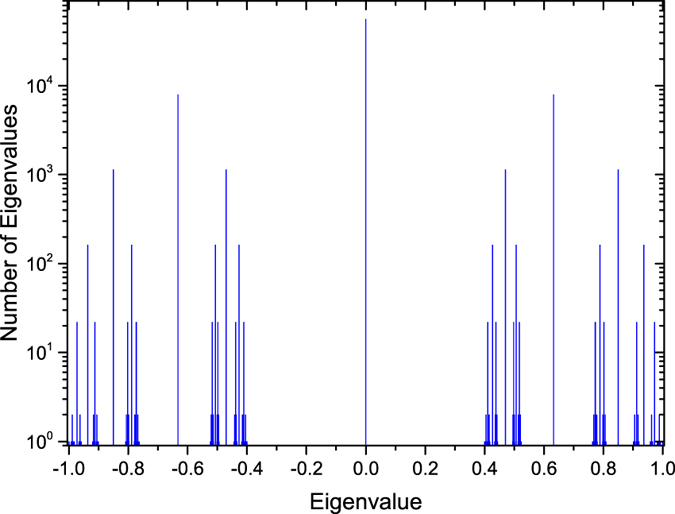
Distribution of distinct eigenvalues for 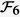 corresponding to *m* = 2 and *δ* = 1.

Since the distribution of eigenvalues conveys much important information, in Fig. 2 we display as a histogram the distribution of eigenvalues for a specific network 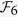 for the case *m* = 2 and *δ* = 1. Because eigenvalues 1, −1, and their offspring are nondegenerate, we only provide the density of eigenvalue 0 and its descendants. Figure 2 indicates that the eigenvalue distribution is heterogeneous.

### Application of eigenvalues

In this section, we apply the obtained eigenvalues and their multiplicities to determine the random target access time for biased random walks and the weighted counting of spanning trees in the weighed scale-free networks 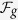. Note that since 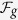 has a treelike structure, the weighted counting of spanning trees is just be the product of weights of all edges in 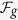. Thus, our aim for evaluating this quantity is to verify that our computation for eigenvalues and their multiplicities is correct.

#### Random target access time

Transition matrix **T**_*g*_ describes the biased discrete-time random walks in 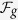, and thus various interesting quantities related to random walks are reflected in eigenvalues of the transition matrix. For example, the sum of reciprocals of 1 minus each eigenvalue (excluding eigenvalue 1 itself) of transition matrix **T**_*g*_ determines the random target access time, also called eigentime identity, in 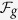
21.

Let *H*_*ij*_(*g*) denote the mean first-passage time from node *i* to node *j* in 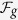, defined as the expected time for a walker starting from node *i* to visit node *j* for the first time. Let 
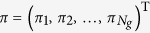
 represent the steady state distribution for random walks on 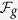
48,49, where 
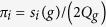
 satisfying 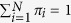 and 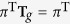. The random target access time, denoted by 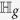, for random walks on 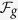, is defined as the expected time needed by a walker from a node *i* to another target node *j*, chosen randomly from all nodes according to the steady state distribution, that is,


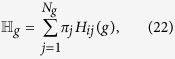


which does not depend on the starting node^20^ and can be recast as


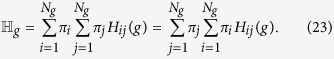


Since 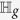 can be looked upon as the average trapping time of a special trapping problem^11^, it encodes much useful information about trapping in 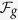.

We introduce a matrix **L**_*g*_ = **I**_*g*_ − **P**_*g*_, where **I**_*g*_ denotes the *N*_*g*_ × *N*_*g*_ identity matrix. Actually, **L**_*g*_ is the normalized Laplacian matrix^23^,^25^,^31^ of 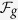. Let *λ*_*i*_(*g*) (1 ≤ *i* ≤ *N*_*g*_) be the *N*_*g*_ eigenvalues of **P**_*g*_. By definition, for any *i*, *σ*_*i*_(*g*) = 1 − *λ*_*i*_(*g*) is an eigenvalue of **L**_*g*_. It can be proved^48^ that 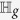 can be represented in terms of the nonzero eigenvalues of **L**_*g*_, given by


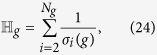


where 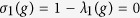 is assumed, with *λ*_1_(*g*) = 1 being the largest non-degenerated eigenvalue of **P**_*g*_.

In Methods, we derive that 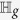 obeys the following recursive relation:


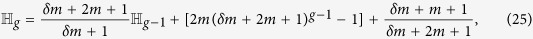


which, with the initial condition 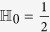, is solved to obtain


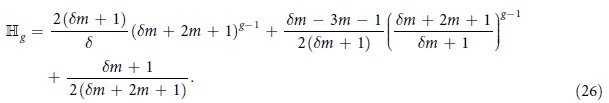



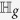
 can be further represented in terms of of network size *N*_*g*_ as


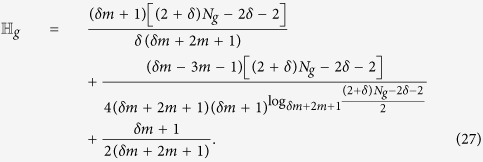


Thus, for large networks (i.e., 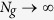), 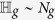, growing linearly with the network size. This is in sharp contrast to that obtained for unweighted scale-free treelike networks^39^ and Cayley tree^36^ (where 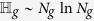), as well as fractal trees (where 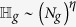 with *η* > 1)^37^,^36^. Thus, the heterogenous distribution of edge weight has a substantial influence on the behavior of random walks in weighted networks.

#### Weighted counting of spanning trees

For a weighted network 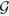, denote by 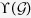 the set of its spanning trees. For a tree 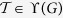, its weight 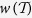 is defined to be the product of weights of all edges *e* in 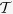, that is, 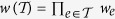, where *w*_*e*_ is the weight of edge *e*. Let 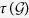 denote the weighted counting of spanning trees of 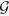, which is defined by 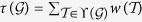.

Since 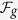 is a tree, it has only one spanning tree, which is in fact 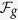 itself. Then, the weighted counting of spanning trees in 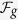 is 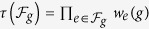, where the product is running over the weight *w*_*e*_(*g*) of all edges 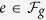. According to previous results^24^, we have


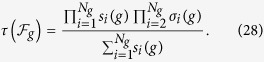


For the sum term in the denominator of Eq. (28), we have


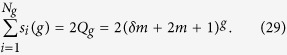


For the two product terms 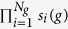 and 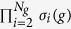 in the numerator of Eq. (28), we use Δ_*g*_ and Λ_*g*_ to represent them, respectively. According to the above-obtained results, the two quantities Δ_*g*_ and Λ_*g*_ obey the following two recursive relations:


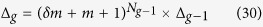


and


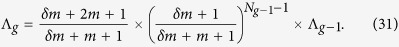


Multiplying Eq. (30) by Eq. (31) results in


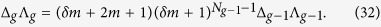


Applying Δ_0_ = 1 and Λ_0_ = 2, Eqs. (32) is solved to give


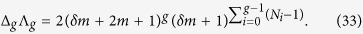


Inserting the results in Eqs. (29) and (33) into Eq. (28) yields


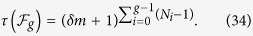


On the other hand, since 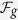 has a treelike structure, 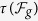 equals the product of weight of all edges in 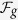. Thus, 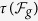 can be directly obtained by evaluating this product. By construction, 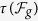 obeys the recursive relation 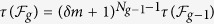. Considering 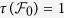, we have 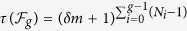, which is consistent with Eq. (34), indicating the validity of our computation on the eigenvalues and their multiplicities for the transition matrix **T**_*g*_ of 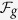.

## Discussion

In conclusion, we have considered the spectra of transition matrix for a class of weight-driven networks, whose degree, strength, and edge weight follow power-law distribution, which is observed in various real-world systems. We have determined all the eigenvalues and their multiplicities of the transition matrix for the networks. Moreover, we have used the obtained eigenvalues to derive a closed-form expression about the random target access time for biased random walks taking place on the networks. Finally, we confirmed our results for the eigenvalues and their multiplicities via enumerating the weighted spanning trees, based on the connection between the two quantities.

We note that although the considered networks look self-similar, they are not topologically fractal. Since many real-life networks are fractal^50^,^51^,^52^, in future it deserves to study the spectra of transition matrix for weighted fractal networks. Furthermore, various structural and dynamical properties of a network are also relevant to the spectra of other matrices^30^, such as adjacency matrix and non-backtracking matrix. Future work should include determining the spectra for adjacency matrix^31^ and non-backtracking matrix^53^,^54^ of weighted scale-free networks.

## Methods

### Proof for the statement that **P**_*β*,*α*_ is a full column rank matrix

Let *v* be an arbitrary vector of order *N*_*g*_ − *N*_*g*−1_:


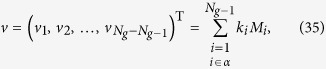


where *M*_*i*_ is the *i*th column vector of **P**_*β*,*α*_ so that 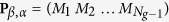. Let 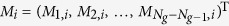. Assume that *v* = 0. Then, we can prove that *k*_*i*_ = 0 holds for arbitrary *k*_*i*_. By construction, for any old node 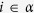, it has a new leaf neighboring node 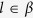. Then, in the expression 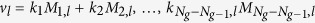, only 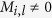, while all *M*_*x*,*l*_ = 0 for *x*≠*i*. From *v*_*l*_ = 0, one obtains *k*_*i*_ = 0. Hence, **P**_*β*,*α*_ is a full column rank matrix.

### Derivation for the recursive relation between 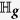 and 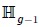


Let 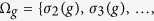 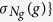
be the set of the *N*_*g*_ − 1 nonzero eigenvalues of matrix **L**_*g*_. For *g* ≥ 1, Ω_*g*_ includes 1, 2, 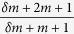, and other eigenvalues generated by them. Thus, Ω_*g*_ can be classified into three nonoverlapping subsets 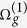, 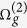 and 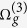, satisfying 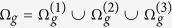, where 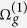 consists of all the 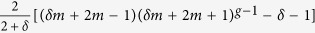 eigenvalues 1, 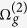 contains only the unique eigenvalue 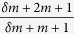, and 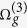 includes those eigenvalues generated by 1, 2, or 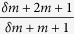. For 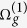 and 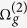, we have 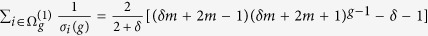 and 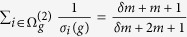. While for 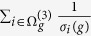, it can be evaluated in the following way.

From Eq. (15), we can derive the following relation dominating the eigenvalues of **L**_*g*_ and **L**_*g*−1_:


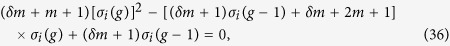


which shows that every eigenvalue 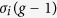 in Ω_*g*−1_ generates two eigenvalues, 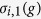 and 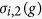, belonging to 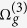. Using Vieta’s formulas, we obtain 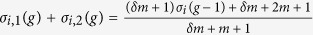 and 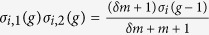. Then


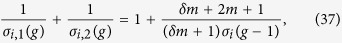


which implies that


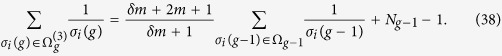


Combining the above-obtained results leads to the following recursive relation between 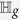 and 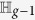:


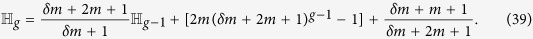


## Additional Information

**How to cite this article**: Zhang, Z. *et al.* Spectra of weighted scale-free networks. *Sci. Rep.* **5**, 17469; doi: 10.1038/srep17469 (2015).
